# The Individual Victim. Hypogeneralization within a New Social Type

**DOI:** 10.1007/s12124-023-09801-z

**Published:** 2023-08-26

**Authors:** Lucas B Mazur

**Affiliations:** 1 Jagiellonian University, ul. Stefana Batorego 12, Kraków, 31-135 Poland; 2 Sigmund Freud University, Berlin, Germany

**Keywords:** Hypogeneralization, Littlefeather, Simmel, Social types, Victimhood, Victimization

## Abstract

Over the past several decades we have seen increased attention paid to victimhood. However, many of these dialogues are only tangentially related to actual experiences of victimization. They are better understood as reflections of a new Simmelian social type, that is, a new pattern emerging within psycho-social interaction—namely, *the Victim*. The current reflections focus on the meaning making processes within these broader discussions of victimhood. We first briefly review Georg Simmel’s understanding of social types and explore *the Victim* as a new social type. We then examine how the process of *hypogeneralization* appears within social types, including *the Victim*, whereby the patterned social interactions that constitute a social type are seen as a single individual or collective. Finally, we examine a recent example from popular discourse of *the hypogeneralized Victim*.

## Introduction

Over the past several decades we have seen increased attention paid to what might be collectively called *victimhood*. However, many of our current victimhood dialogues are only tangentially related to actual experiences of victimization. They are better understood as reflections of a new Simmelian social type, that is, a new pattern emerging within psycho-social interaction—namely, *the Victim*. Thus, rather than placing our primary focus on the psychological antecedents or consequences of experiences of victimization, we will focus on the meaning making processes within these broader discussions of victimhood. We will first briefly review Georg Simmel’s understanding of social types and explore *the Victim* as a new social type. We will then examine how the process of *hypogeneralization* (as explained within the political context by Carriere, 2022) appears within social types, including *the Victim*, whereby the patterned social interactions that constitute a social type are seen as a single individual or collective. Finally, we will examine a recent example from popular discourse of *the hypogeneralized Victim*.

## Simmelian Social Types and *the Victim*

For Georg Simmel, social types are patterns, or forms, that appear before us within the swirl of social interaction, including social discourse (for a discussion of social types and related concepts such a social role and social figure, see Almog, 1998 and le Grand, 2019). We find ourselves and our interaction partners appearing within these forms; they afford us or deny us various ways of psycho-social being in the world. More particularly, social forms are found at the intersection of *non-conflicting dichotomies*, such as how *the Stranger* is, according to Simmel (1950), a form of social interaction that is both: distant *and* near, invested in the Other *and* disinterested in the Other, known *and* unknown, coming *and* going, etc. Importantly, no side cancels the other out; they both exist in what might be thought of as a kind of balance, despite their apparent mutual exclusivity. In other words, within the Simmelian dialectic there is no resolution of these tensions (as there is for Hegel). Each social type (e.g., *the Poor*, *the Miser*, *the Adventurer*) is constituted by a particular combination of such non-conflicting dichotomies. While social types necessarily appear as particular people or peoples, their form is not dependent on any particular people or peoples, which is also to say that no individual or collective is the given social type in any sort of stable, let alone essentialized, sense. Rather, it is as if we slide in and out the given “role” within the ongoing ebb and flow of social interaction, again, depending upon the presence or absence of balance between the relevant non-conflicting dichotomies. If one “side” of a constituent dichotomy in effect outweighs or otherwise cancels the other side, the social type vanishes,. for example, as seen when *the Stranger*, who necessarily both comes and goes, “buys land”—thereby becoming too wedded to the given locale and thus too “local” (Simmel, 1950). While people and peoples pass in and out of any given social type, the form of the social type remains; floating, as it were, as a recognizable possibility along the waves of our social interactions, now to appear here, then to appear there.

Recently, we have seen the appearance of a new social type, *the Victim* (for a richer presentation than can be provided here, see Mazur, 2023). This new social type has arisen in the wake of various societal changes. For example, we have observed a fairly recent shift in moral cultures away from *honor culture* and *dignity culture*, to what has been called *victimhood culture*, wherein the recognition of victimhood confers moral superiority (Campbell & Manning, 2018). Otto Marquard (1991) noticed something similar in the growing presence of what he called *Tribunalization*, which might be summarized as an ever-growing, watchful, judging eye before which we must all plead our innocence, convincing others and ourselves that our ways of being are justified and not harmful towards others. This sustained attention to harm has been further nurtured by the expanding culture of positivity, in which everyone must “feel good” at all times and be watchful of any cracks in our smile (Illouz, 2008). In other words, within our increasingly therapeutic culture, we have grown increasingly sensitive and responsive to negativity (Sugarman & Martin, 2018). The presence of this watchful eye has only spread further via the “look-at-me culture” of the internet and social media, in which we are placed before the unknown masses for judgment and in which we ourselves judge unknown others. Victimhood culture, especially in the digital age, involves appeals to (primarily unknown) third parties. Judgement hangs in the air, and all must be justified and “positive.” It is in this context that *the Victim* has emerged.

Similar to the disconnect between *the Poor* as a social type and experiences of poverty (Simmel, 1965), *the Victim* is only tangentially related to experiences of victimization. What is of more central importance to the social type is the balance struck between particular non-conflicting dichotomies that emerge within the wider context mentioned above. We can now briefly explore six such non-conflicting dichotomies that constitute *the Victim*. While victimization itself necessarily speaks to some form of weakness, (1) *the Victim* is both weak and strong. While acknowledging the weakness contained within victimhood, when it comes to *the Victim* we also think of various kinds of strength, e.g., moral righteousness, the valor of the “underdog.” For example, while *the Victim* may be physically defeated, *the Victim* can be seen as morally superior. (2) *The Victim* is different from us, but *the Victim* is also the same as us; difference is both celebrated and denied. We deny difference, understanding perceived difference as a form of aggression, while we also celebrate difference, seeing it as a reflection of the authentic self (which we want to acknowledge). (3) *The Victim* is a status that horrifies, but it is also a status that is desired. We see this in such notions as “competitive victimhood,” whereby people and peoples who lament victimhood in effect compete to be seen as the most victimized (Young & Sullivan, 2016). (4) *The Victim* is neither too temporally distant nor too temporally close. *The Victim* is found in the past, the present, and the future, but importantly, not at any one time alone. While victimization can be identified at fixed points in time—as going on now, as having taken place in the past, and/or as a threat arising in the future—*the Victim* necessarily combines all three times. Something has taken place in the past that is of continued relevance today and which can only be truly addressed at a point in the perennial “tomorrow.” It is a way of being, not a problem to be solved in the present, a danger on the horizon to be averted, or a past sin for which we are to repent and for which we might forgive or be forgiven. Thus, (5) *the Victim* is unhealable, voiding apology and denying forgiveness. Finally, (6) *the Victim* is not dependent on the particular experiences of any particular individual or group. The stability of its form is afforded it by the fungibility of its exemplars. Similarly to how *the Poor* exists in all societies and even all social and economic classes irrespective of the particulars of the people living there (Simmel, 1965), *the Victim* can appear anywhere. Just as *the Poor* is sustained by aid institutions whose existence involves the perpetuation of the social type irrespective of the particular poor people to whom they provide assistance, *the Victim* is increasingly supported by institutions (e.g., organized around such keywords as trauma, equity, diversity, and inclusion; e.g., Wong, 2023) irrespective of the particulars of anyone’s actual victimization.

## Hypogeneralization

Hypogeneralization takes place when an individual, or a collective, is understood as encapsulating and speaking with a unified voice to a singular understanding of a much larger, more complex issue. As part of the emphasis he places on the role of the individual within a cultural psychology of politics, Carriere (2022, p. 44–45) describes how hypogeneralization can galvanize support for, or opposition to, a particular understanding of particular policy positions. For example, when discussing immigration, politicians often focus on the experiences of a single immigrant or immigrant group. In other words, they tell a singular story, and this story helps shape how voters perceive the larger issue. The story not only helps to direct and focus people’s understanding of the matter, orienting them within the overwhelming complexity of the larger issue, but that narrative can become the rallying point around which this encapsulated understanding can gain momentum. What is more, this singular narrative can come to be so attached to the individual that the particular vision of the broader issue comes to be seen as that person; the individual is understood not only as a good example of the position, but as embodying the position, as *being* the position. Carriere (2022) describes how particular wealthy and powerful individuals can be seen as in effect *embodying* wealth and power. Intelligence has become encapsulated as *Einstein*, evil and cruelty as *Stalin* or *Hitler*, betrayal as *Benedict Arnold*, and caring and compassion as *Mother Teresa*. Our attempts to overcome the ultimately insurmountable limits of humanity have been told in myth as the story of *Icarus*, and more recently we see this expressed within the fictional name *Frankenstein*. These are perhaps trite examples, but as Carriere (2022) observes, the psychological process of hypogeneralization that helps to create them is commonplace.

## The Hypogeneralization of Simmelian Social Types

In as far as we are able to perceive particular social types as patterns emerging within the swirl of social interaction, seeing a form persist despite changes to the particular content in which it finds expression, we might argue that we already have the beginnings of a form of hypogeneralization. This gestalt-like process is what allows us to speak in terms of social types in the first place, even giving them particular names, e.g., *the Miser*, *the Adventurer*, *the Poor,* and now *the Victim*. Simmel’s social types are of an emergent nature, which is to say that their forms arise from within the psycho-social complexities of social interactions (including social discourses). A pattern emerges within the non-conflicting dichotomies as the particular form of the given social type, and when that form is filled with the particular content (i.e., a particular people or person in a particular context at a particular time), we are able to look out at the world and point with our finger saying, “there it is.”

Thus, not only the form comes to be seen as crystalized, but the content does as well, which is to say that particular people or peoples come to be seen as *being the Miser*, *the Adventurer*, *the Poor*, etc. We can briefly illustrate this using Simmel’s (1950) understanding of *the Stranger*. The balance of distance and nearness, of caring and indifference, etc., found within *the Stranger* afford us a unique openness to, and before, others. The openness of the patient in psychotherapy, much like that of the penitent in confession, is possible to the extent that there is the balance of *the Stranger* between patient and therapist, penitent and priest, and that openness can be threatened by the loss of that balance (e.g., by becoming too close or too distant). The balance affords us unique ways of being (e.g., unique candor, even about the most private and personal of matters), which are perceived as a repeated pattern of interaction, a pattern which can then be seen as synonymous with a particular context or person. The therapist or therapy, the priest or confession, can come to be seen as *being* the “strangeness” that affords such openness. Similarly, Simmel (1950) describes how particular groups, in particular contexts and at particular times, come to be seen as *being the Stranger*. However, while social types necessarily appear in particular contexts or in the shape of particular people or peoples, their appearance in the world not only comes and goes, but it can shift between people, peoples, and contexts. The person or people seen as *the Stranger* today (or any other social type for that matter), need not be so perceived tomorrow. We might say that in as far as we see a social type within the particulars of its expression, we are exhibiting a form of hypogeneralization; not only are larger patterns perceived within social interaction, but they are “named,” and then the particular people or peoples that embody the social type come to be seen as actually *being* those types. While not entirely unrelated to experiences of strangeness, poverty, adventure, etc., it is important to repeat that social types are only superficially related to those experiences that bear the same name. Particular people are seen as embodying the type not due to their own experiences (of poverty, strangeness, victimization, etc.), but rather as hypogeneralized expressions of stabilized patterns seen within social interaction.

## Hypogeneralization of the Victim and the Example of Sacheen Littlefeather

Like all social types, *the Victim* can be hypogeneralized; coming to be seen as in effect *being* a particular person or people. Within such hypogeneralization, the individual becomes an embodiment of the social type and the focus shifts away from their individuality and onto the ways in which they represent the balance between the various non-conflicting dichotomies that constitute the given social type. When it comes to the hypogeneralization of *the Victim*, that would include such non-conflicting dichotomies as weakness / strength, sameness / difference, desired / abhorred, temporal distance / temporal proximity, the institutionalization / of individual experiences, the apology / for the unforgivable.

An example of *the hypogeneralized Victim* can be seen in the social discourses that emerged in 2022 around the refusal of the Best Actor award by Marlon Brando, the public statement of which was made at the 1973 Oscars by Maria (or Marie) Louise Cruz, better known as Sacheen Littlefeather. In declining the award, Brando explained his decision as a public protest against the ways in which Native Americans were portrayed and treated by the film industry (“Hollywood”), and to draw attention to the coeval standoff at Wounded Knee involving the American Indian Movement (AIM) and the Federal Bureau of Investigation (FBI). While the particulars of the gesture were widely discussed from the beginning, particularly given the public debates about the standoff between AIM and the FBI at Wounded Knee taking place at that time, what is more interesting for our current purposes is the manner in which the matter reappeared in the public consciousness in 2022 in the form of a public apology to Sacheen Littlefeather by the Academy Awards and a televised celebration thereof on September 17, 2022 called *An Evening with Sacheen Littlefeather*. Not only do we see *the Victim* emerge, but we see Littlefeather in effect become the hypogeneralized version of that social type. In such a short piece as this, our summary of this example must necessarily be brief, but it should suffice to show how this particular person came to be seen as the embodiment of *the Victim*, as the hypogeneralized representation of the social type, which itself is a singularized form, a perceived pattern, of social interaction that appears within the greater complexity and dynamism of our psycho-social lives.

The tripartite temporal nature of the apology is nicely illustrated in the following statement taken from the letter from the President of the Academy of Motion Pictures Arts and Sciences, David Rubin, to Littlefeather:

Today, nearly 50 years later, and with the guidance of the Academy’s Indigenous Alliance, we are firm in our commitment to ensuring indigenous voices—the original storytellers—are visible, respected contributors to the global film community. We are dedicated to fostering a more inclusive, respectful industry that leverages a balance of art and activism to be a driving force for progress. (Sachdeva, 2022)

Here we see resolve in the present, “today,” arising out of a recognition of the past, the resolution of which will take place in a future of (ever-forward-driven) progress. It is not that we have learned from the past and that we are somehow improved today, but that we are honoring and learning from the past today, and will do better tomorrow. The author of the letter concludes by asserting that Littlefeather is “forever respectfully engrained in our history,” again, a forward-moving recognition of the past made in the present. *The Victim* is neither too temporally distant (either in the past or the future), nor too temporally close (in the present).

The author of the letter also mixes acknowledgments of weakness with the recognition of strength. For example, he writes about the “abuse [she] endured because of this statement” (at the Oscars) and about how her career suffered as a result, while he also calls her statement “powerful” and worthy of “sincere admiration.” Her story is told as that of a brave and strong woman, who also needed to be protected. For example, in earlier versions of the story she was the target of verbal insults backstage, which in later versions became her needing the protection of several people from an inevitable physical attack by John Wayne (Hiltzik, 2022; Nehme, 2022). Given Wayne’s numerous well-known roles in Westerns, it is worth noting how these stories contain echoes of “Cowboys and Indians.”

When refusing the award in 1973 on behalf of Brando, Littlefeather wore clothing of buckskin, and she wore her waist-length hair straight and bound on the sides in “pigtails.” Littlefeather herself recounted Brando’s request that she dress the part of a Native American when refusing the award (“he chose my wardrobe for me”; Academy Awards, 2022, 57:50). She was to stand out as “different” at the award ceremony and yet her traditional beauty, which was and remains much commented on (Littlefeather worked as an actress and model even before 1973), made that difference blend easily into the familiar. What might partially account for the “familiarity” of her look, and even the “familiarity” of her beauty, is that according to genealogical research and Littlefeather’s own sisters, their family was not Native American (Keeler, 2022; Kreps, 2022). Their mother’s family was Dutch, French, and German, while her father was of Spanish-Mexican decent with no tribal identity. Thus, difference and similarity are both celebrated and denied. Similarly, in the letter Rubin writes that we are all to be part of the Academy Award’s mission to “inspire imagination and connect the world through cinema,” while we are also told that this mission is impossible without Native Americans, whom he calls the “original storytellers,” a setting apart of certainly one of the most universal features of all human cultures. The familiar is rendered strange, and the commonplace, unique.

The historical treatment of Native American peoples is heartbreaking and appalling. However, within *the Victim* that same status is also desirable. We see this within how Littlefeather’s story has been told and retold. While very few people, if anyone, would actually want to become the target of pubic disapprobation, condemnation, or worse, public martyrdom for a just cause is admired, applauded, and even desired by many (even if only hypothetically). The mixture of boos *and* applause that greeted Littlefeather’s reading of Brando’s statement constitute a position from which we shrink *and* that we envy.

Brando’s public statement made at the 1973 Oscars came to be ascribed to Littlefeather—certainly by 2022—precisely as part of the hypogeneralization of *the Victim*. Importantly, if the public refusal of the award were to be more strongly associated with Brando, the dialogue around it would not as easy strike the required balance within the non-conflicting dichotomies required of *the Victim*. Brando, especially in light of his film persona and personal stardom, is *too strong* and *too familiar* (e.g., clearly representing “dominant” groups). He *cannot be seen as himself being in an abhorred position*, but rather, with his success and fame most would see him as being in a solely enviable one. As a representative of Hollywood, for many one of *the* representatives of Hollywood, his gesture would too clearly position the industry on the side of the “good,” in effect shifting the industry from foe to ally. It would be an act of public penance, washing away sin, something which is not possible within *the Victim*. His protest, in refusing this concrete award at this particular time, is also *too locked in time* (i.e., the moment of the protest), and the gesture does not as easily slip into the past or continuously into the nebulous future. By contrast, when seen as a gesture from Littlefeather, the award itself, as well as its refusal, in effect vanish from the conversation. In sum, the Academy is not responding in 2022 to Brando’s gesture in 1973, but to the continuity afforded by the person of Littlefeather. More precisely, they are responding to Littlefeather as a hypogeneralized expression of *the Victim*. To be clear, they are not responding to the individual person who was Sacheen Littlefeather, but to the individualized vision of *the Victim*. This is also why the “apology,” the televised celebration put online for all to see, the institutionalization of the apology, etc., can sustain their value even in light of the news that Littlefeather was not Native American, in light of the fact that the gesture was actually that of Brando, etc. The social type is more durable than the particulars in which it materializes. If not her, someone else can easily be found. The form of a social type cannot be denied by the undermining of a single manifestation.

## Conclusion

This brief piece first provided theoretical reflections on the nature of the new social type known as *the Victim*. In the spirit of Georg Simmel, particular attention was paid to the six non-conflicting dichotomies constitutive of *the Victim*: (1) weakness / strength, (2) sameness / difference, (3) desired / abhorred, (4) temporal distance / temporal proximity, (5) the institutionalization / of personal experiences, and (6) apology / for the unforgivable. We then examined how *hypogeneralization* plays an important role for social types in general, and for *the Victim* in particular. Hypogeneralization speaks to the increasing singularity with which social types are perceived, first as a repeating, recognizable shape within the swirl of social interaction, including discourse, and then as a particular person or people perceived to embody the type. In this way, *the individual Victim* can walk among us—this, despite the fact that no person or people can constitute the social type in any kind of essentialized manner. This was illustrated with the example of Sacheen Littlefeather and the Academy Awards.

This new social type is a powerful theoretical and analytical tool with which we might better understand contemporary discourses related to victimhood. In line with the thinking of Simmel, this new social type is not only of academic interest, but also attests to an important psycho-social phenomenon of increasing social prominence and that thus constitutes an important aspect of our lives. What is more, a better understanding of this new social type can help us to better understand the similarities and differences between victimization, victims, victimhood, and *the Victim*.
